# Medical students and house officers’ perception, attitude and potential barriers towards artificial intelligence in Egypt, cross sectional survey

**DOI:** 10.1186/s12909-024-06201-8

**Published:** 2024-10-31

**Authors:** Rasha Mahmoud Allam, Dalia Abdelfatah, Marwa Ibrahim Mahfouz Khalil, Mohamed Mahmoud Elsaieed, Eman D. El Desouky

**Affiliations:** 1 Cancer Epidemiology & Biostatistics Department, National Cancer Institute, Cairo University, Cairo, Egypt; 2 Nursing Faculty, Alexandria University, Alexandria, Egypt; 3 Medical Student at Kasr Alainy Medical School, Cairo University, Cairo, Egypt

**Keywords:** Artificial intelligence, Attitude, Barriers, House officers, Medical students, Perception

## Abstract

**Background:**

Artificial intelligence (AI) is one of the sectors of medical research that is expanding the fastest right now in healthcare. AI has rapidly advanced in the field of medicine, helping to treat a variety of illnesses and reducing the number of diagnostic and follow-up errors.

**Objective:**

This study aims to assess the perception and attitude towards artificial intelligence (AI) among medical students & house officers in Egypt.

**Methods:**

An online cross-sectional study was done using a questionnaire on the Google Form website. The survey collected demographic data and explored participants’ perception, attitude & potential barriers towards AI.

**Results:**

There are 1,346 responses from Egyptian medical students (25.8%) & house officers (74.2%). Most participants have inadequate perception (76.4%) about the importance and usage of AI in the medical field, while the majority (87.4%) have a negative attitude. Multivariate analysis revealed that age is the only independent predictor of AI perception (AOR = 1.07, 95% CI 1.01–1.13). However, perception level and gender are both independent predictors of attitude towards AI (AOR = 1.93, 95% CI 1.37–2.74 & AOR = 1.80, 95% CI 1.30–2.49, respectively).

**Conclusion:**

The study found that medical students and house officers in Egypt have an overall negative attitude towards the integration of AI technologies in healthcare. Despite the potential benefits of AI-driven digital medicine, most respondents expressed concerns about the practical application of these technologies in the clinical setting. The current study highlights the need to address the concerns of medical students and house officers towards AI integration in Egypt. A multi-pronged approach, including education, targeted training, and addressing specific concerns, is necessary to facilitate the wider adoption of AI-enabled healthcare.

**Table:** 

Contributions to the literature
• Recent years have seen a rise in the application of artificial intelligence in the medical field. Nevertheless, Egypt is among the nations lacking in various AI implementation techniques in the medical field
• According to study participants, lack of awareness, disinterest in the field, inadequate training, lack of a curriculum, low financial resources, and a lack of technological advancements in our country are major reasons of failure of AI implementation in Egypt
• Thus, it is mandatory that AI implementation techniques must be held in the medical field as they could be of great help in diagnosis and treatment

## Background and rationale

Artificial intelligence (AI) is a software that utilizes data sources to assist humans in decision-making [1, 2]. Recent advancements in AI have significantly role, aiding in the treatment of various illnesses and reducing diagnostic errors [3–5]. Research has demonstrated the potential benefits of AI algorithms across multiple medical fields, including ophthalmology [6], dermatology [7], and pathology [8]. High-income countries have made substantial financial investments in AI research, particularly within the medical sector [9].

Despite its potential, there are misconceptions regarding AI’s capabilities and its future role in healthcare. Common assumptions include the belief that AI will replace doctors, and that effective use of AI necessitates programming knowledge. Incorporating AI into medical education is essential, as it can provide personalized feedback to help students understand AI algorithms. Furthermore, AI has various applications in public health, such as detecting disease outbreaks through social media and search engine query data. Notably, the Chinese Academy of Sciences modeled the COVID-19 outbreak using multi-source information fusion, while Google predicted influenza epidemics [10–12].

AI-powered computer tools will assist primary care providers in accurately identifying patients who demand further attention and delivering tailored regimens for everyone. Primary care physicians have the ability to utilize artificial intelligence (AI) to record their notes, evaluate their conversations with patients, and input necessary data directly into Electronic Health Record Systems (EHRS). These programs will gather and examine patient data and deliver it to primary care physicians, along with valuable information about the patient’s medical requirements. To ensure that the medical workforce can effectively employ emerging technologies, it is imperative for medical schools and organizations that facilitate ongoing medical education to promptly develop and implement this approach. Efforts have already been made to enhance the overall ethical consciousness of individuals involved in the development of AI technology. Prominent educational institutions and research centers have now incorporated ethics into their technical curricula, specifically aiming to enhance the abilities of developers, programmers, and engineers to engage in critical thinking [13].

Studies indicate that medical students and healthcare professionals often possess limited knowledge and understanding of AI, its capabilities, and its potential applications in healthcare. This knowledge gap may hinder the responsible adoption of AI technologies [14–16]. Attitudes and perceptions towards AI in healthcare vary significantly among medical students and professionals. While some view AI as a transformative technology that can enhance patient outcomes, others express concerns regarding the reliability, security, and ethical implications of AI-based medical decision-making [17].

Past research has identified various barriers that can impede the widespread adoption of AI in healthcare, such as concerns about liability, lack of regulatory guidelines, resistance to change, and the need for more robust educational and training programs to prepare healthcare professionals to work with AI systems [18]. To ensure that the medical workforce can effectively utilize new technologies, it is crucial for continuing medical education providers and medical schools to develop and implement relevant curricula promptly [19]. Integrating AI-based content into medical education will be essential, given the rapid pace of technological advancement alongside biomedical knowledge. Preparing teachers to teach various parts of AI technologies is crucial [20]. Therefore, we conducted this research to investigate medical students’ perceptions and attitudes towards artificial intelligence in Egypt.

### Study hypothesis

Medical students and house officers in Egypt possess diverse perceptions and attitudes towards artificial intelligence in healthcare, with significant variations based on their educational background, level of exposure to AI technologies, and awareness of AI applications. Furthermore, several barriers, including insufficient training, and lack of awareness, lack of resources limit integration of AI into medical practice.

## Materials and methods

### Research design

Cross-sectional study.

### Target population

The target population is Egyptian medical students and house officers. The study was conducted from the 14th of May to the 10th of October 2023.

### Sample size determination

The sample size estimation was based on a previous study by Swed et al. (2022), which indicated that approximately 70% of medical students have prior knowledge of AI [21]. To achieve a two-sided 99.99% confidence interval for a single proportion, we used the large sample normal approximation, extending 5% from the expected proportion. This calculation indicated a required sample size of 1027 participants. To account for potential refusal to participate, we increased the sample size by 30%, resulting in a total requirement of 1335 participants. The sample size was calculated using the Epi Info statistical package [22]. This rigorous estimation ensures that our findings are statistically robust and reflective of the target population.

### Sampling strategy

The participants were recruited through a combination of a convenience and snowball sampling technique. Data were collected through an online survey, where participants were recruited and completed the questionnaire using a Google Form. The researchers then instructed the initial participants to share the survey link to their respective class group through various social media platforms (WhatsApp, Instagram, Facebook, and Twitter), allowing the sample to expand through the snowball sampling approach.

### Statistical analysis

The data management and analysis were conducted using the Statistical Package for Social Sciences (SPSS) version 27. The numerical data were summarized using either means and standard deviations or medians and/or ranges, as appropriate. Numerical data were explored for normality using Kolmogrov-Smirnov test and Shapiro-Wilk test. Comparisons between two groups for not normally distributed numeric variables were done using the Mann Whitney U test. The categorical data were summarized using frequency and percentages. The Cronbach’s α coefficient was employed to evaluate the scale’s internal consistency. The Chi-Square test was used to compare groups of categorical data. Ultimately, a logistic regression model and stepwise selection were employed to predict the artificial intelligence outcome measurements, specifically the perception and attitude, based on the initial features of the study population. The unadjusted odds ratios and their related 95% confidence intervals were utilized in the regression analysis. A p-value of 0.05 or lower was deemed to be statistically significant. The tests were conducted using a two-tailed approach.

### Data collection instruments

A web-based survey using social media apps (WhatsApp, Facebook, and Messenger) and email that was in accordance with the Checklist for Reporting Results of Internet E-survey (CHERRIES) [23].

The research was carried out following the Checklist for Reporting Results of Internet E Surveys guidelines for the online survey and the Strengthening the Reporting of Observational Studies in Epidemiology (STROBE) Statement guidelines for reporting observational studies. The participants were told briefly about the research, how to fill in the questionnaires and the period needed to complete it, after which an online informed consent was taken. Participation in the study was voluntary.

## Instrument for data collection

Questions used in these sections were adopted from available literature [21, 24].

The research survey consists of three parts:

Part I: Socio-demographic characteristics: Age, gender, medical specialty and academic year.

Part II: participants’ perception about AI domain. it consists of a total of ten items (the questions were calculated with a total perception score of 10 and a value of 1 for each right response and a value of 0 for each wrong answer). Perception questions include (perception about usage of AI in diagnosis via diagnosis algorithm, radiological diagnosis, pathological diagnosis, treatment decision, treatment using AI robots, patients treating themselves, improving drug research, supporting personalized medicine, nursing procedures and nursing diagnosis). Bloom’s original cutoff points were used to judge perception as good (> 75%), moderate (50–75%), or poor (< 50%). Overall, we considered participants who scored less than 50% with an inadequate perception, while those who scored 50–100% had an adequate perception [25].

Eight questions with checkboxes (Multiple answers could be chosen) about possible advantages of AI in medicine, and six questions about disadvantage of AI, finally six questions about potential barriers and other questions about concerns about AI, AI previous courses or education and self-evaluation of the level of knowledge and source of that knowledge. One question about the expected sector to use it, another two questions about possible risk: students were asked which problems they are concerned about regarding the application of AI in medicine. It is not clear who is liable when there are adverse clinical outcomes between humans and AI; therefore, we included a question about liability for AI decisions in medicine.

Part III: Questions about participants’ attitude about AI, the attitudes of students consist of a total of 13 closed-ended questions with options of strongly disagree, disagree, don’t know, agree, and strongly agree. Attitude score was calculated by assigning a value of 1 to strongly disagree, 2 for disagree, 3 for don’t know, 4 for agree and 5 for strongly agree with total score range from (13 to 65). Cut-off of 80% was used to determine sufficient attitude (≥ 80%), and positive attitude (≥ 52 out of 65) [26].

## Data quality control

The questionnaire was first developed in the English language and afterwards translated into Arabic. To ensure the reliability and consistency of the data collecting instrument, it was then translated back into English.

The validity of the expertise judgements related to the standard precautions perception questions and compliance was assessed by three experts in the area of public health. The three experts agreed with and validated the content of those knowledge and compliance questions, as they found the survey items to be appropriate and valid as initially developed.

A pilot study was conducted on 15 participants to assess clarity, usability, technical performance, applicability, and response to the questionnaire; no changes to the survey were required. The pilot test was conducted independently, and the current study does not incorporate the results. Subsequently, the validity and reliability of the questionnaire were confirmed through a pilot study involving 50 participants. The final number of survey items did not differ significantly from the initial set of items compiled through the literature review and cognitive interviews. After the pilot study with 15 participants, implying the survey content and structure was deemed appropriate and did not need modification. The expert validation and pilot testing processes confirmed the validity and reliability of the survey, leading to the final version being largely consistent with the initially developed items.

Cronbach’s alpha values demonstrated the internal consistency of the employed sub-scales in the tool, it was 0.82 for the whole questionnaire ranging from 0.6 to 0.82 (perception = 0.6, and Attitude = 0.81). Inclusion criteria were responders being medical students and house officers. Exclusion criteria were non-medical responders and an incomplete survey.

## Results

### Demographic data of study participants

The current study included 1346 medical students. The average age of participants was 21 ± 2 years. Greater than have of participants were females (57%). Most of the participants were undergraduates (74.2%) while around a quarter of them were house officers (25.8%), Regarding year of study, students from various years of study were included; where 26.1% (*n* = 261) were from the 2th year, 20.6% (*n* = 206) from the 3th year and the rest were more or less equally distributed between the remaining studying years (Table 1).

**Table 1:** Characteristics of study participants

	*n* = 1346 (%)
**Age (years) (Mean ± SD)**	21 ± 2
**Sex**	
Male	579 (43)
Female	767 (57)
**Qualification level**	
Medical students	999 (74.2)
House officers	347 (25.8)
**Academic year (n = 999)**	
1st	172 (17.2)
2nd	261 (26.1)
3rd	206 (20.6)
4th	136 (13.6)
5th	127 (12.7)
6th	97 (9.7)

SD: Standard deviation

### Usage of AI in health care & legal problems

Table (2) shows that specialized clinics and university hospitals are the most important health care sectors to commercialize artificial intelligence. Most participants (55.3%) stated that the company that created AI will be liable for legal problems caused by usage of AI. One third of participants (29.8%) stated that the rate of legal problems will be high after the usage of AI.

**Table 2:** Usage of artificial intelligence in health care & legal problems

	*n* = 1346 (%)
The first health care sector to commercialize artificial intelligence	
Public primary care such as public health centers	227 (16.9)
Primary care in private clinics	288 (21.4)
Specialized clinics (spine, knee, obstetrics and gynecology, etc.)	406 (30.2)
University hospitals	400 (29.7)
Others	25 (1.9)
**Who do you think will be liable for legal problems caused by artificial intelligence?**	
Doctor in charge	381 (28.3)
Company that created the Artificial Intelligence	745 (55.3)
Patient who consented to follow Artificial Intelligence’s input	220 (16.3)
**How do you think the liability for legal problems caused by artificial intelligence?**	
Very low	102 (7.6)
Low	269 (20)
Same rate as a traditional practice	342 (25.4)
High	401 (29.8)
Very high	232 (17.2)

### Personal knowledge about AI and source of knowledge

As shown in Table (3), the majority of the participants (78.3%) thought that they are knowledgeable about AI, half of them (48.5%) reported that their knowledge about AI is basic understanding and greater percentage of them (66.9%) reported that their knowledge about AI was self-taught. The majority of participants (84.8%) reported that, they have never attended any courses about AI. If the doctor’s judgment and an artificial intelligence’s judgments differ, most of the participants (63.5%) reported that it is better to follow doctor opinion.

**Table 3:** Personal knowledge about AI and source of knowledge

	*n* = 1346 (%)
Overall, in your opinion do you think you are knowledgeable about artificial intelligence	
No	292 (21.7)
Yes	1054 (78.3)
**Artificial intelligence knowledge level** ***If yes*** **(n = 1054)**	
Basic understanding	511 (48.5)
Working knowledge	284 (26.9)
Relevant skills in AI	233 (22.1)
Proficient in AI	26 (2.5)
**Artificial intelligence knowledge source** ***If yes*** **(n = 1054)**	
Self-taught	705 (66.9)
Learned by attending educational courses	205 (19.4)
Learned from work-related activities	144 (13.7)
**Attending a course on Artificial intelligence**	
Never	1141 (84.8)
This year	105 (7.8)
Last year	57 (4.2)
Two to three years ago	28 (2.1)
More than three years ago	15 (1.1)
**If the doctor judgment and an artificial intelligence’s judgments differ**, **which will you follow?**	
Doctor’s opinion	855 (63.5)
Artificial intelligence’s opinion	111 (8.2)
Patients’ choice	380 (28.2)

## Knowledge about advantages, disadvantages of AI and potential barriers

Table (4) shows possible advantages and disadvantages of AI in the medical field. As AI can analyze large amount of relevant data, greater than half of participants (56.9%) state that AI is important in the medical field. Meanwhile, most participants (64.7%) state that lack of emotions and empathy is a major disadvantage of AI. Concerning barriers that prevent the usage of AI in medicine in Egypt. The majority of participants (64.3%) stated that the most important barrier that faces AI, is lack of financial resources.

**Table 4:** Knowledge about artificial intelligence advantages, disadvantages & barriers to practice in medical field

Artificial intelligence advantages	Yes *n* (%)	No *n* (%)
Analyzing large amounts of clinically relevant data	766 (56.9)	580 (43.1)
Speeding up processes in health care	658 (48.9)	688 (51.1)
Making more accurate treatment decisions	423 (31.4)	923 (68.6)
Reducing medical errors	645 (47.9)	701 (52.1)
Improving the cost-effectiveness of medicine	542 (40.3)	804 (59.7)
Giving doctors more time discussions and clinical examinations	464 (34.5)	882 (65.5)
Does not get tired and can work 24 h	688 (51.1)	658 (48.9)
Having no emotional exhaustion nor physical limitation	627 (46.6)	719 (53.4)
**Artificial intelligence disadvantages**		
Being not flexible enough to be used for every patient	736 (54.7)	610 (45.3)
Can amplify biases that already exist in data sets and lead to patient discrimination	385 (28.6)	961 (71.4)
Can undermine the autonomy of patients	449 (33.4)	897 (66.6)
lacking the ability to develop empathy and consider the patient’s emotional well-being	871 (64.7)	475 (35.3)
Can be developed by programmers with little experience in medical practice	603 (44.8)	743 (55.2)
Causing uncertainty about who is liable if something goes wrong	601 (44.7)	745 (55.3)
**Barriers to practice**		
lack of interest	506 (37.6)	840 (62.4)
lack of awareness	754 (56.0)	592 (44.0)
lack of proper training	827 (61.4)	519 (38.6)
lack of curriculum	538 (40.0)	808 (60.0)
lack of financial resources	866 (64.3)	480 (35.7)
lack of technological advancement	849 (63.1)	497 (36.9)

### Artificial intelligence perception & attitude

**Fig. 1:**
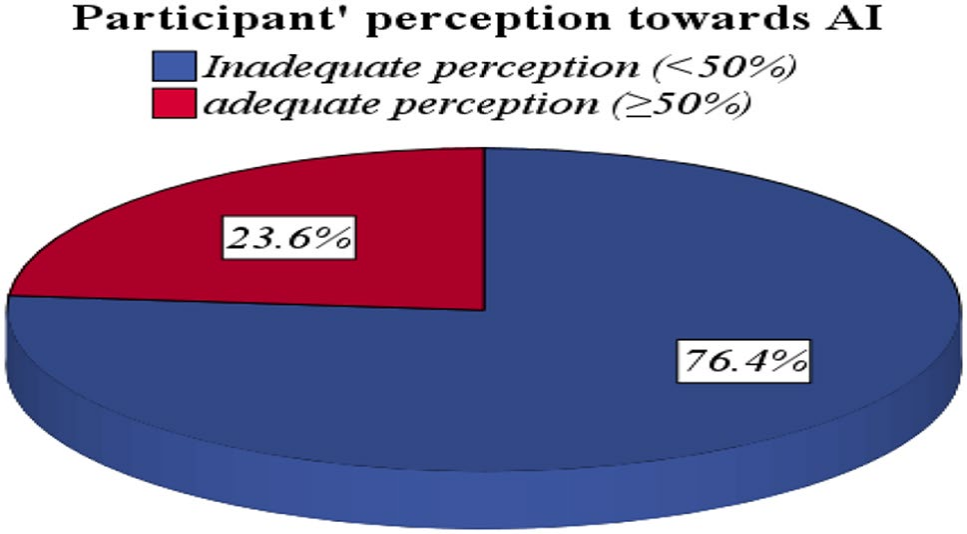
Participants’ perception towards Artificial intelligence

**Fig. 2:**
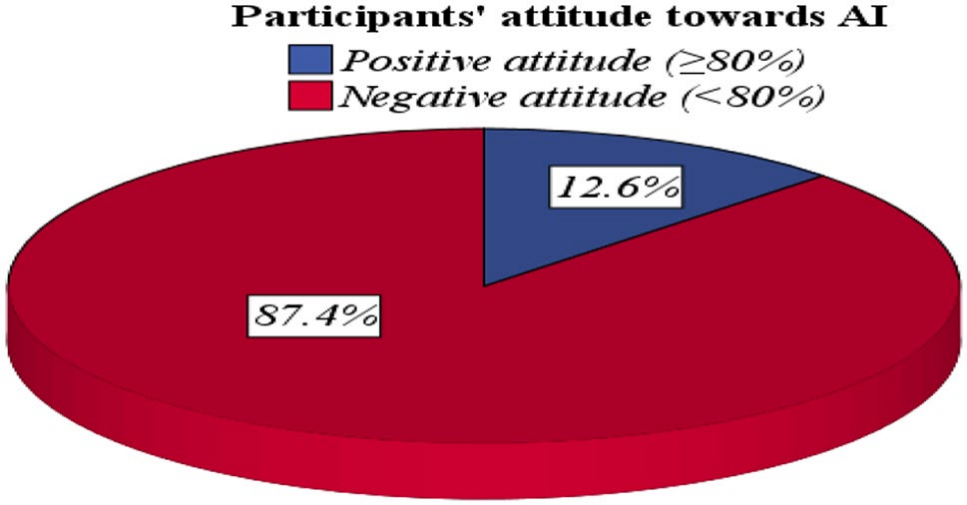
Participants’ attitude towards Artificial intelligence

Table (5) shows perception assessment about usage of artificial intelligence in medical field. The average AI perception score was 3.2 ± 2.1 ranging from (0–9). Most participants have inadequate perception (76.4%) about the importance and usage of AI in the medical field, while only (19.8% & 3.8%) have moderate and good perception, respectively (Fig. 1). Regarding attitude, the average attitude score was 44.2 ± 7.9 ranging from (13–65). The majority of the participants (87.4%) have a negative attitude, while only (12.6%) of participants have a positive attitude (Fig. 2). The current study shows that, there is no statistically significant difference in AI perception and attitude score among students and house officers (p-value 0.057&0.183, respectively) (Table 6). 

**Table 5:** Perception & attitude towards Artificial intelligence of study participants

Perception score	*n* = 1346 (%)
Mean ± SD	3.2 ± 2.1
Adequate (≥ 50%)	317 (23.6)
Inadequate (< 50%)	1029 (76.4)
**Attitude score**	
Mean ± SD	44.2 ± 7.9
Positive (≥ 80%)	170 (12.6)
Negative (< 80%)	1176 (87.4)

SD: Standard deviation

**Table 6:** Perception and attitude score towards AI among medical students and house officers

	Medical students		House officers	
	Median (range)	Median (range)		*p* value
Perception score	3 (0–9)		3 (0–9)	0.057
Attitude score	45 (13–65)		45 (16–65)	0.183

### Factors associated with artificial intelligence perception & attitude

Table (7) shows the relation of AI perception and attitude to different factors. There was no statistically significant difference in level of perception concerning (gender, qualification level & academic year). Regarding how attitudes toward AI can differ depending on baseline variables, there is statistically significant difference between males and females regarding the proportion of positive attitude (p-value < 0.001). Also, there is statistically significant difference in proportion of positive attitude in relation to the specific medical specialty (p-value = 0.039) and specific academic year (p-value = 0.024) with higher attitude among second year students (16.1%) compared to fifth year students (5.5%). It was remarkable that great percentage of participants with adequate perception have positive attitude (18.9%) compared to participants without adequate perception (10.7%).

**Table 7:** Perception & attitude score classification in relation to different factors

	Adequate perception (≥ 50%)	Inadequate perception (< 50%)		Positive attitude (≥ 80%)	Negative attitude (< 80%)	
	*n* = 317 (%)	*n* = 1029 (%)	*P* value	*n* = 170 (%)	*n* = 1176 (%)	*P* value
**Age (years) (Mean ± SD)**	21.49 ± 2.31	21.17 ± 2.19	0.025	20.94 ± 2.03	21.28 ± 2.25	0.090
**Sex**						
Male	142 (24.5)	437 (75.5)	0.476	95 (16.4)	484 (83.6)	< 0.001
Female	175 (22.8)	592 (77.2)		75 (9.8)	692 (90.2)	
**Medical specialty**						
Dentistry	11 (25)	33 (75)	< 0.001	7 (15.9)	37 (84.1)	0.039
Medicine	114 (27)	309 (73)		37 (8.7)	386 (91.3)	
Nursing	146 (20.2)	577 (79.8)		109 (15.1)	614 (84.9)	
Pharmacy	13 (17.6)	61 (82.4)		10 (13.5)	64 (86.5)	
Physiotherapy	31 (46.3)	36 (53.7)		5 (7.5)	62 (92.5)	
Veterinary medicine	2 (13.3)	13 (86.7)		2 (13.3)	13 (86.7)	
**Qualification level**						
Medical students	242 (24.2)	757 (75.8)	0.340	122 (12.2)	877 (87.8)	0.453
House officers	75 (21.6)	272 (78.4)		48 (13.8)	299 (86.2)	
**Academic year (n = 999)**						
1st	28 (16.3)	144 (83.7)	0.052	18 (10.5)	154 (89.5)	0.024
2nd	74 (28.4)	187 (71.6)		42 (16.1)	219 (83.9)	
3rd	44 (21.4)	162 (78.6)		30 (14.6)	176 (85.4)	
4th	38 (27.9)	98 (72.1)		18 (13.2)	118 (86.8)	
5th	31 (24.4)	96 (75.6)		7 (5.5)	120 (94.5)	
6th	27 (27.8)	70 (72.2)		7 (7.2)	90 (92.8)	
**Perception towards AI**						
Inadequate (< 50%)				110 (10.7)	919 (89.3)	< 0.001
Adequate (≥ 50%)				60 (18.9)	257 (81.1)	

To measure the independent effect of different covariates on degree of perception and attitude toward AI, factors which had significance level less than 0.100 were selected to enter stepwise logistic regression analysis. Multivariate analysis revealed that age is the only independent predictor of AI perception (AOR = 1.07, 95% CI 1.01–1.13). However, perception level and gender are both independent predictors of attitude towards AI (AOR = 1.93, 95% CI 1.37–2.74 & AOR = 1.80, 95% CI 1.30–2.49, respectively), as shown in Table (8)

**Table 8:** Multivariate analysis

	B	S.E.	*P* value	AOR&95% CI for OR
Perception towards Artificial intelligence				
Age (years)	0.06	0.03	0.024	1.07 (1.01–1.13)
**Attitude towards Artificial intelligence**				
Artificial intelligence perception level	0.66	0.18	< 0.001	1.94 (1.37–2.74)
Sex	0.59	0.17	< 0.001	1.80 (1.30–2.49)

B: regression coefficient, SE: standard error, AOR; adjusted odds ratio, CI: confidence interval

## Discussion

AI is growing into the public health sector and is going to have a major impact on every aspect of primary care, so the inclusion of AI teaching in medical curricula has been advocated in several publications. This seems to be one of the first studies to explore the general opinions of medical students regarding artificial intelligence in medicine, achieving a remarkable 98% response rate from one of the largest samples of medical students in our country. Our findings reveal a relatively low overall perception of AI and its applications in healthcare among surveyed students and house officers. The limited coverage of AI and its healthcare applications within the current medical education curriculum in Egypt may have contributed to the observed knowledge gaps among the students. Formal training and exposure to AI-related topics may be lacking, hindering the students’ understanding of this emerging technology. Furthermore, medical students and healthcare professionals in Egypt may have limited access to up-to-date resources, research, and case studies showcasing the practical applications of AI in healthcare. This lack of exposure could have influenced their perceptions and understanding of the technology’s potential benefits and drawbacks. Cultural attitudes towards technology within Egyptian society can further shape medical students’ and professionals’ views on AI. Resistance to change, influenced by traditional practices, may create barriers to adopting AI innovations. Concerns about job displacement and the erosion of human oversight in clinical decision-making are prevalent among students, reflecting a broader apprehension regarding the ethical implications of AI in sensitive healthcare contexts. Moreover, the lack of opportunities for hands-on experience with AI tools may exacerbate students’ knowledge gaps and reluctance to embrace AI integration in healthcare. Without practical exposure, students may struggle to see the relevance and potential of AI in enhancing patient care .

The current study involves 1346 participants, 999 of them were medical students (74.2%) and the rest were house officers this is consistent with study by Swed et al. (2022) [21] on 1,494 medical students and doctors in Syria, 1,252 (83.1%) are undergraduate medical students while 255 (16.9%) are doctors. Regarding gender distribution in our study, we found that nearly half of the participants were females (57%), lower female percentage were reported by Pinto dos Santos et al. (2019) [27], Bisdas et al. (2021) [28], Wood et al. (2021) [29] & Al Saad et al. (2022) [30], (36.9%, 45%, 47.8%, 49.27% &53.8%, respectively). Regarding year of study as reported by Al Saad et al. (2022) [30], the highest percentage was in favor of the 6th year (31.8%), followed by the 5th year (7.8%) and the rest were equally distributed between the remaining studying years, however the highest percentage was in favor of the 2nd year (26.1%), followed by the 3rd year (20.6%) in our study. This discrepancy may indicate a unique sampling strategy or a specific interest in the views of earlier-year students regarding AI, possibly reflecting a growing awareness and engagement with technology in medical education at an earlier stage in their training and highlighting the importance of ensuring that all years of study incorporate AI training, especially in earlier years, to build a foundational understanding before students reach clinical practice.

In our study, 205 medical students (19.4%) reported having attended a course on artificial intelligence (AI). This figure aligns closely with the findings of Al Saad et al. (2022), indicating a consistent level of exposure to AI education among medical students in Egypt [30]. However, it surpasses the results from Sit et al. (2020), where only 9% of UK medical students had received any form of AI instruction, highlighting a potentially more progressive approach to AI integration in Egyptian medical education [31] (Figs. 1 and 2).

The current study found that the number of medical students attended a course on artificial intelligence was 205 (19.4%) this was more or less the same as the results of Al Saad et al. (2022) [30], but higher than finding of A study by Sit et al. (2020) [31], on UK medical students towards AI only 45 (9%) received some form of teaching in AI, In terms of training and education in AI, Santos et al. (2021) [32], Indicated that the acquisition of AI knowledge primarily occurred through self-directed learning (41%) and job-related experiences (25%). Participating in classes and pursuing postgraduate training accounted for only 12% and 5% of the respondents, respectively. It is possible that the reason for this is the insufficient inclusion of AI education and training in past and present medical physics education programs. Compared to our results, self-teaching accounting for 66.9%, work-related activities for 13.7%, and attending educational courses for 19.4%. However, half of the study participants (48.5%) reported that their knowledge about AI is basic understanding, this agrees with Wood et al. (2021) [29] & Al Saad et al. (2022) [30] results (48.4% & 47%, respectively). On the contrary Al Ahmari et al. (2022) [33] study reported that the majority of participants (77.1%) lack basic knowledge of the working principles of artificial intelligence. The current study reported that about quarter of participants (26.9%) reported that their perception about AI is working perception which is lower than what was stated by Santos et al. (2021) (34%) [32].

Our study revealed that only 38% of participants had a basic knowledge of artificial intelligence (AI), which aligns with findings from other research [24, 27, 29, 34]. This level of awareness is significantly lower than the 71.3% reported by Ahmed et al. (2022) in Pakistan, indicating regional disparities in AI education and understanding among medical students [35]. Such differences may reflect variations in curriculum focus, access to resources, and the overall integration of AI into medical training across different countries. The current lack of familiarity with AI technologies among medical educators further complicates this issue. Many educators report feeling inexperienced and confused about how to effectively teach AI concepts, which can hinder the development of comprehensive educational programs. To address these challenges, it is essential to establish skilled, multidisciplinary teams within medical education that are well-versed in the real-world applications of AI. These teams can facilitate better training and integration of AI into curricula, ultimately aiming for improved health outcomes [29]. Regarding gender differences, our findings indicated no statistically significant difference in AI perception between male and female participants. This finding is consistent with the results reported by Swed et al. (2022) [21]. However, contrasting results from Pakistan showed that a majority of those with AI knowledge were male [35]. This discrepancy may suggest cultural or educational factors that influence the engagement of different genders with AI topics, highlighting the need for inclusive educational strategies that encourage participation from all demographics.

Interestingly, more than half of our participants (53.9%) recognized the crucial role of AI in diagnostics, particularly through diagnostic algorithms. This indicates an understanding of AI’s potential impact on clinical practice. Furthermore, 32.6% of participants strongly affirmed that AI is utilized in radiology, although a study in Pakistan reported that 56.7% of students viewed AI as essential to radiology practice [35]. The variation in these perceptions may result from differences in exposure to AI applications in local healthcare settings and the prevalence of AI technologies in various medical fields.

This study identified that about one quarter of individuals agree (25.8%), and only (4.4%) strongly agree that physicians will eventually be replaced by AI. A cross-sectional survey conducted in Syria revealed near results, indicating that (21.5%) of participants believe that AI could do that [21], the same results was concluded from the study of Pinto dos Santos et al. (2019) [27], which revealed that (96.6%) of students disapproved of the claim that physicians in general could be replaced in the near future, also study by Perrier et al. (2022) [36], who reported only 6% of participants expressed their fear of losing their job because of AI and the same low proportion was also found in study by Polesie et al. (2020) [37] (5.4%). Compared to the findings of the Pakistan study by Ahmed et al. (2022) [30], where the majority of participating medical students view artificial intelligence (AI) as a helpful tool for healthcare workers rather than alternative to physicians, the finding in our study may be the result of misconceptions about the potential benefits and limitations of AI, however study by Doumat et al. (2022) [38], reported higher percentage of agreement (55.8%) that in the near future, AI will replace some human specialists.

Before adopting new technologies, people need to consider they are advantageous and simple to use. Studies by Doumat et al. (2022) & McLennan et al. (2022) [38, 39], noted that the analysis of substantial large amounts of clinically relevant data is one of the most frequently reported benefits of employing AI in medicine. (95.4% & 95.6, respectively), the same findings stated by Oh et al. (2019) [24], who agreed that the area of medicine in which AI would be most useful is disease diagnosis (83.4%), this in concordance with our study findings (56.9%), also Ejaz et al. (2022) [40] concluded that the majority of participants believed that artificial intelligence would be crucial to the provision of healthcare services in the future (74.4%), also the current study reported that approximately one-third of participants don’t think AI will lead to a rise in medical errors. The percentage of participants in Pakistan who agreed that using AI in medicine will lower diagnostic errors was doubled to 66.6% [35]. Our study reported that the least reported advantages of using AI in medicine is making more accurate treatment decisions (31.4%), however the least reported AI disadvantage in the study by Oh et al. (2019) [24] was that; AI has no emotional exhaustion or physical limitation (0.4%).

Regarding AI disadvantages McLennan et al. (2022) [39], concluded that the most frequently mentioned drawback is that there isn’t enough information to use it as a guide in unforeseen circumstances. (85.9%), however study by Oh et al. (2019) [24] reported that the possible problem cited by the participants was that; it is not flexible enough to be applied to every patient (34.1%), however, the most frequently mentioned disadvantage in our study was the inability to develop empathy and take the patient’s emotional well-being into consideration (85.9% versus 64.7%). The least addressed disadvantage of applying AI to medicine is that it may exacerbate preexisting biases in data sets and result in patient discrimination (28.6%), however the least reported AI disadvantage in the study by Oh et al. (2019) [24] was that AI has no emotional exhaustion or physical limitation (0.4%). We believe that proper education about the potential use of AI and its limitations should be presented to students in a clear way and AI should be compulsorily integrated into the medical school curriculum to avoid this misconception.

The findings from our study reveal significant barriers to the integration of artificial intelligence (AI) in medical practice, with a notable 64.3% of participants reporting a lack of financial resources and 63.1% pointing to insufficient technological advancement. This is consistent with Singh et al. (2023), who identified a lack of awareness (66.7%) as the primary obstacle, followed by inadequate college training and technical resources (55.4%) [41]. These barriers highlight the need for targeted interventions to enhance awareness and training regarding AI in the medical community.

Regarding the inclusion of AI in the curriculum of medical schools, (24.4%) strongly agree & (57.5%) agree that AI should be included in curriculum of medical and nursing schools, which is also consistent with Pakistan’s findings that stated that (21.1%) strongly agree and (53%) agree [35]. AI technologies need to be taught in the context of a comprehensive, integrated curriculum by interdisciplinary educational teams [42–44]. It will take time and flexibility to incorporate AI-based materials into medical education because technology is changing equally quickly as knowledge about biology. Preparing educators to teach different facets of AI technologies is important [20].

In the current study, the average perception score about AI was 3.2 ± 2.1, and only 23.5% of medical students were of moderate to good perception, similar results concluded by Fritsch et al. (2022) [45] that reported about 24.0% of participants were of good perception, lower score values were reported by Swed et al. (2022) [21], that concluded that the mean score of Knowledge of AI among doctors and medical students in Syria was 1.82 ± 1.83 and (16.5%) of participants were of good knowledge, also Pinto dos Santos et al. (2019) [27] showed that German students had an overall low level of knowledge about AI, with students stating that they acquired this from mainstream media rather than university teaching. It is important that we offer our future medical professionals with sufficient educational opportunities, and they must be able to use computers and software in a way that facilitates the practice of evidence-based medicine [31, 46]. On the other hand, approximately half of the participants stated that they have enough knowledge about overall AI applications, in a study by Sit et al. (2020) [31] & Brandes et al. (2020) [47] (47.1&50%, respectively).

Regarding AI perception relation to different factors on univariate level we found that only statistically significant related factors to AI perception are age and medical specialty, higher mean age is associated with adequate perception, also physiotherapy and medicine students have the highest AI perception (46.3% and 27%, respectively), prediction of adequate Knowledge of AI among the study population depend on the variables which were significant on univariate level. Our results showed that the only independent predictor factor influencing the AI perception was age, in contrast to the results by Swed et al. (2022) [21], It was found that medical students’ level is the only independent predictor of AI perception.

Regarding attitudes toward AI, our study reported an average attitude score of 44.2 ± 7.9, with 55.2% of participants demonstrating a positive attitude. This is slightly lower than the findings by Swed et al. (2022), who reported a mean attitude score of 6.03 ± 2.16, with 68.7% of participants expressing a positive attitude toward AI [21]. Furthermore, studies by Perrier et al. (2022) and Coppola et al. (2021) noted even higher levels of positive attitudes, with 86% and 77% respectively favoring the implementation of AI tools in their specialties [36, 48]. These variations in attitude scores may reflect differences in study populations, educational contexts, and the specific AI applications discussed. The relatively moderate positive attitude toward AI in our study suggests that while there is a foundation of support for AI integration, there remains a need for enhanced education with AI technologies among medical students. The findings of our study regarding attitudes toward artificial intelligence (AI) reveal significant associations with various demographic factors, including gender, medical specialty, undergraduate year, and AI perception level. Notably, second-year medical students exhibited the highest positive attitude toward AI (16.1%), while fifth-year students demonstrated the lowest (5.5%). This may suggest that younger students are more engaged with research and innovations, including AI, during their training, with integration of these technologies into clinical practice. Gender difference was also evident, with a higher percentage of males (16.4%) reporting a positive attitude compared to females (9.8%). This aligns with the findings of Swed et al. (2022), which indicated that more men (37.4%) held a positive attitude toward AI than women (30.7%) [21]. Such gender disparities may reflect broader societal attitudes toward technology and innovation.

Among specialties, physiotherapy and medicine students exhibited the highest positive attitudes (92.5% and 91.3%, respectively). This may be attributed to the relevance of AI technologies in their fields, particularly in areas like rehabilitation and diagnostics, where AI can enhance patient outcomes. Additionally, our study found a relation between higher levels of AI perception and more positive attitudes. This reinforces the idea that as students become more knowledgeable about AI, their attitudes toward its integration in healthcare improve. This finding is consistent with Swed et al. (2022), who also noted that baseline characteristics, such as gender and year of study, significantly influence attitudes toward AI [21]. Comparatively, the results regarding undergraduate year reflect some differences with other studies. For instance, while our study found second-year students to have the most positive attitudes, Syrian medical students in Swed et al. (2022) reported that sixth-year students had the highest positive attitudes (26.4%). This suggests variability in attitudes may be influenced by specific educational environments and exposure to AI during different training periods. Furthermore, Park et al. (2021) found that first-year medical students were more likely than fourth-year students to believe that AI would significantly impact medicine [49]. Conversely, Doumat et al. (2022) reported no statistically significant difference in attitude scores between pre-clinical and clinical years, which aligns with our findings [38].

Since the willingness to accept innovation and raise awareness of AI applications in modern medicine are issues that must be addressed, the future of medicine and healthcare in Egypt will increasingly rely on artificial intelligence (AI), so it is advised that a suitable AI curriculum be developed and put into place. Decision makers should aim to develop policies to bring about the innovations of AI technology in the medical field. The results of this study indicate that there is a need to improve the current knowledge and perceptions of AI among medical students and healthcare professionals in Egypt. The data highlights the relatively low levels of understanding and mixed attitudes towards the potential of AI in the medical field within this population.

To address this, we recommend that immediate actions be taken to better prepare Egyptian medical students and healthcare professionals for the evolving role of AI in medicine. This should include providing targeted training courses and seminars to enhance their perception and influence their attitudes towards the responsible and effective utilization of AI-based technologies. Additionally, we suggest that professional educators incorporate discussions and hands-on exposure to AI applications within the medical curriculum to better equip the future healthcare workforce.

By investing in these educational and training initiatives, we can help ensure that Egyptian medical students and professionals are well-informed and equipped to navigate the patient-centered digital future of healthcare.

## Conclusions and recommendations

The study found that medical students and house officers in Egypt have an overall negative attitude towards the integration of AI technologies in healthcare. Despite the potential benefits of AI-driven digital medicine, most respondents expressed concerns about the practical application of these technologies in the clinical setting. study highlights the need to address the concerns and apprehensions of medical students and house officers towards AI integration in Egypt, rather than assuming automatic acceptance of these technologies. A multi-pronged approach, including education, targeted training, and addressing specific concerns, is necessary to facilitate the wider adoption of AI-enabled healthcare.

The integration of artificial intelligence (AI) into medical field, particularly in the public health sector, require improvement of perception of medical students and staff toward AI through adding AI to curriculum of medical students and continuous education of medical staff through workshops and seminars on AI usage and applications.

### Limitations & strengths

Even though it is inexpensive and practical, across-sectional research methodology is not currently able to prove causality. However, this study’s applicability was strengthened by large sample size and a high response rate. Use a validated instrument, account for potential confounders in the final model, and draw a sample from different research locations to improve the internal validity of the results. To make sure the study is reliable, priori sample size computations are also carried out.

The cross-sectional design of our study limits our ability to examine changes over time or establish causal relationships between variables. While this design is effective for assessing the current perceptions and attitudes of medical students towards AI, it does not account for how these perceptions may evolve as exposure to AI technologies increases or as medical education adapts. Future research could benefit from a longitudinal approach, allowing for the observation of trends over time and the assessment of how educational interventions or technological advancements impact students’ understanding and attitudes towards AI. This could provide more robust data on the effectiveness of AI integration in medical curricula.

## Data Availability

Data that support the findings of this study have been deposited in google sheet with link:https://docs.google.com/spreadsheets/d/1FLFysgSCNhgS8xM6Q0jJLolleg77NjjNv2hEwOSDkjo/edit?usp=sharing.

## Abbreviations

AI: Artificial intelligence
AOR: Adjusted odds ratio
B: Regression coefficient
CHERRIES: Checklist for Reporting Results of Internet E-survey
CI: Confidence interval
EHRS: Electronic Health Record Systems
SD: Standard deviation
SE: Standard error
SPSS: Statistical Package for Social Sciences
UK: United Kingdom
WHO: World Health Organization
